# Supplementary figures and images for: Cross-protocol assessment of induction and durability of VISP/R in HIV preventive vaccine trial participants
Source: PLOS Glob Public Health. 2023 Jun 8;3(6):e0002037. doi: 10.1371/journal.pgph.0002037 (PMC10249892; doi:10.1371/journal.pgph.0002037)

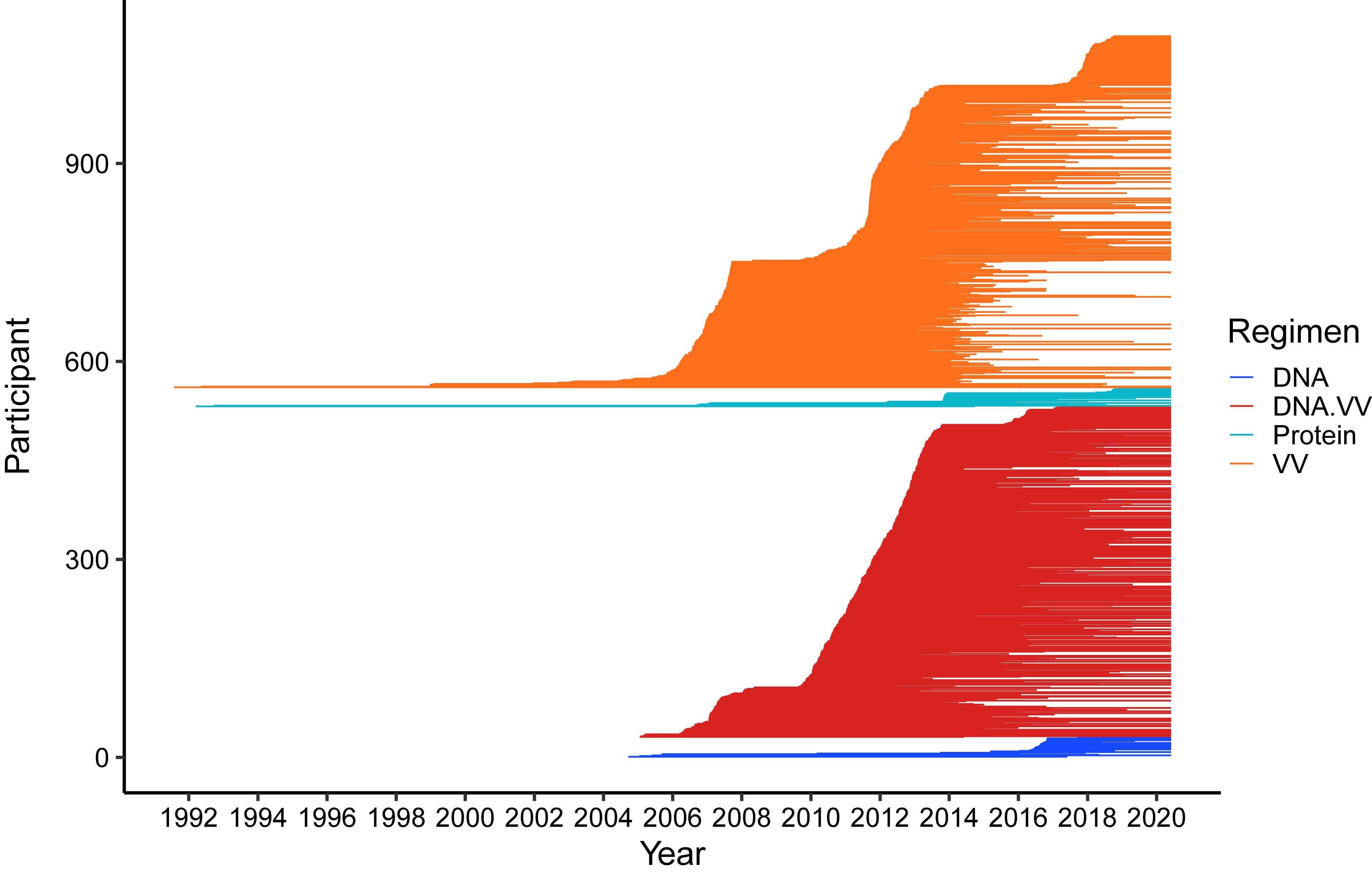

Supplement: S1 Fig — Participants enrolled in HVTN 910 were monitored for HIV infection during the active study period and monitored for HIV infection and VISP/R during the HVTN 910 active study period. Time of first VISP/R is assumed as date of last HIV vaccination, and participants were followed up until VISP/R resolution, loss to follow up, or study termination, whichever comes first. (TIF) [file pgph.0002037.s001.tif]

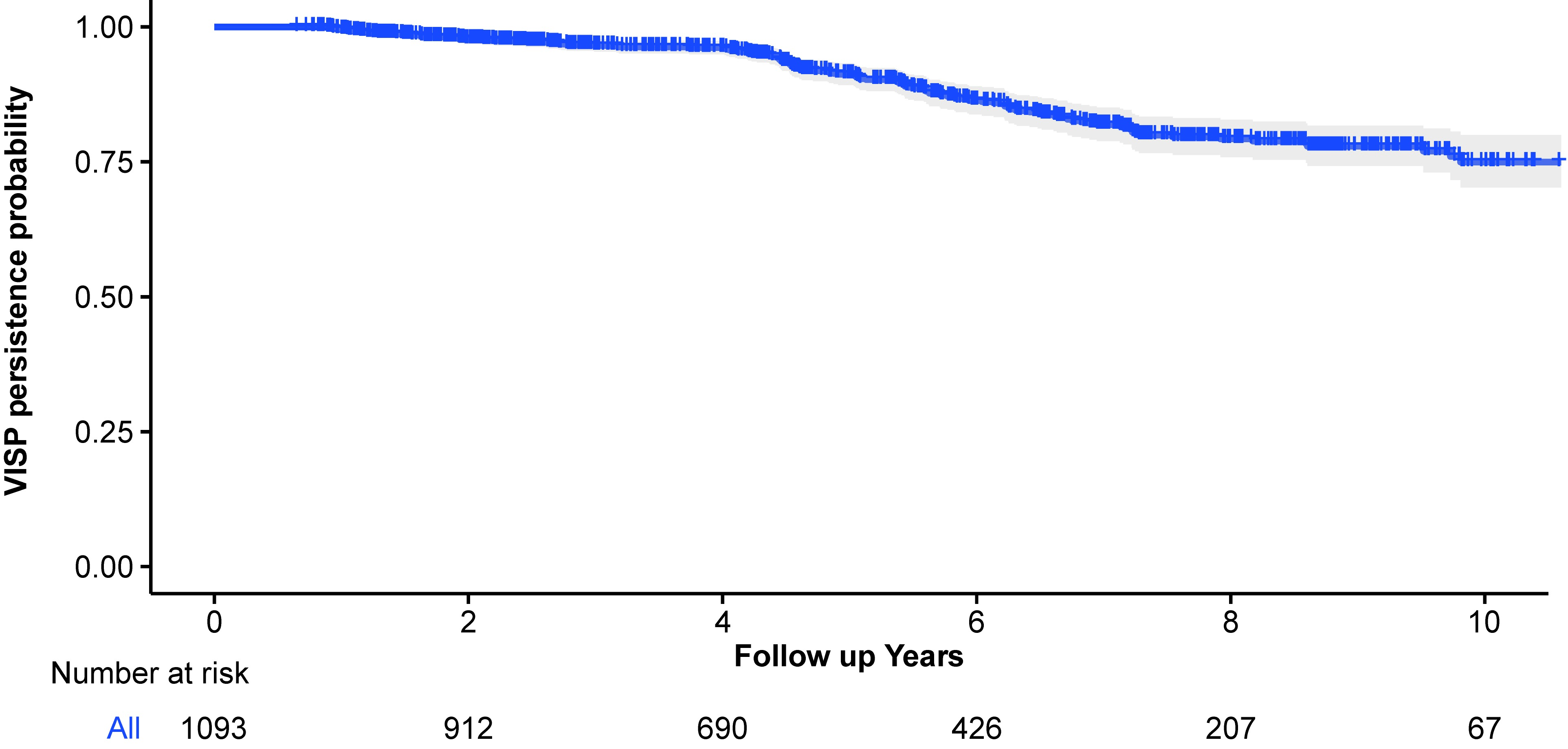

Supplement: S2 Fig — Kaplan-Meier (KM) estimates of all HVTN 910 Participants; shaded regions represent 95% CIs. Participants were censored at time of their per-protocol VISP/R resolution. “At risk” refers to number of participants observed at each timepoint that can contribute to the estimation of VISP/R persistence. (TIF) [file pgph.0002037.s002.tif]
