# Supplementary material for: Cross-protocol assessment of induction and durability of VISP/R in HIV preventive vaccine trial participants
Source: PLOS Glob Public Health. 2023 Jun 8;3(6):e0002037. doi: 10.1371/journal.pgph.0002037 (PMC10249892; doi:10.1371/journal.pgph.0002037)
Supplement: S1 Table — (DOCX) [file pgph.0002037.s003.docx]

**S1 Table: Multivariate analysis of VISP/R rate.**

| **Category** | VISP rate (%) | 95% CI | Multivariate Odd Ratio | P value |
| --- | --- | --- | --- | --- |
| **Demographics** |  |  |  |  |
| **Age** |  |  |  |  |
| 30 or above | 1706/3096 = 55.1% | (53.3%, 56.9%) | Ref | *<*0.001 |
| Less than 30 | 2584/5059 = 51.1% | (49.7%, 52.4%) | 1.196 |  |
| **Sex assigned at birth** |  |  |  |  |
| Female | 1550/3301 = 47.0% | (45.3%, 48.7%) | Ref | 0.335 |
| Male | 2740/4854 = 56.4% | (55.0%, 57.8%) | 1.053 |  |
| **Study Phase** |  |  |  |  |
| Phase 1-2a | 2502/5208 = 48.0% | (46.7%, 49.4%) | Ref | 0.001 |
| Phase 2b | 1788/2947 = 60.7% | (58.9%, 62.4%) | 1.204 |  |
| **Region** |  |  |  |  |
| Sub-Saharan Africa | 522/1950 = 26.8% | (24.9%, 28.8%) | Ref | *<*0.001 |
| Americas/Europe/WP | 3768/6205 = 60.7% | (59.5%, 61.9%) | 4.478 |  |
| **Vaccine Platform** |  |  |  |  |
| DNA | 177/1213 = 14.6% | (12.7%, 16.7%) | Ref | *<*0.001 |
| DNA.VV | 1874/2426 = 77.2% | (75.5%, 78.9%) | 17.919 |  |
| Protein | 103/216 = 47.7% | (41.1%, 54.3%) | 3.227 |  |
| VV | 2136/4300 = 49.7% | (48.2%, 51.2%) | 7.326 |  |
| **Gag** |  |  |  |  |
| No gag | 3853/7432 = 51.8% | (50.7%, 53.0%) | Ref | *<*0.001 |
| gag | 437/723 = 60.4% | (56.8%, 63.9%) | 1.634 |  |
