# Supplementary material for: Cross-protocol assessment of induction and durability of VISP/R in HIV preventive vaccine trial participants
Source: PLOS Glob Public Health. 2023 Jun 8;3(6):e0002037. doi: 10.1371/journal.pgph.0002037 (PMC10249892; doi:10.1371/journal.pgph.0002037)
Supplement: S2 Table — (DOCX) [file pgph.0002037.s004.docx]

**S2 Table: Binding antibody titers and VISP/R**

| Category | Obs | Coef | Odds Ratio | Lower CI | Upper CI | P-value |
| --- | --- | --- | --- | --- | --- | --- |
| Con 6 gp120/B response: Y vs N | 920 | 1.218 | 3.379 | 1.322 | 8.641 | 0.011 |
| Age Less than 30 vs Age great or equal to 30 | 920 | -1.178 | 0.308 | 0.205 | 0.462 | <0.001 |
| Sex Male vs Female | 920 | -0.25 | 0.779 | 0.533 | 1.138 | 0.197 |
| Gag vs Non-gag | 920 | -0.841 | 0.431 | 0.043 | 4.297 | 0.473 |
| DNA.VV vs DNA | 920 | 5.967 | 390.439 | 138.824 | 1098.099 | <0.001 |
| Protein vs DNA | 920 | 1.58 | 4.855 | 0.346 | 68.06 | 0.241 |
| VV vs DNA | 920 | 3.464 | 31.958 | 18.419 | 55.448 | <0.001 |
|  |  |  |  |  |  |  |
| Category | Obs | Coef | Odds Ratio | Lower CI | Upper CI | P-value |
| Con S gp140 CFI response: Y or N | 876 | 0.575 | 1.777 | 0.406 | 7.771 | 0.445 |
| Age Less than 30 vs Age great or equal to 30 | 876 | -1.153 | 0.316 | 0.214 | 0.466 | <0.001 |
| Sex Male vs Female | 876 | -0.335 | 0.715 | 0.499 | 1.026 | 0.069 |
| Gag vs Non-gag | 876 | 1.05 | 2.857 | 0.242 | 33.682 | 0.404 |
| DNA.VV vs DNA | 876 | 3.982 | 53.647 | 14.633 | 196.673 | <0.001 |
| Protein vs DNA | 876 | 2.069 | 7.916 | 0.612 | 102.34 | 0.113 |
| VV vs DNA | 876 | 3.483 | 32.569 | 18.775 | 56.497 | <0.001 |
|  |  |  |  |  |  |  |
| Category | Obs | Coef | Odds Ratio | Lower CI | Upper CI | P-value |
| gp70B.CaseAV1V2 response: Y or N | 892 | 1.526 | 4.598 | 2.803 | 7.542 | <0.001 |
| Age Less than 30 vs Age great or equal to 30 | 892 | -1.445 | 0.236 | 0.151 | 0.368 | <0.001 |
| Sex Male vs Female | 892 | -0.333 | 0.717 | 0.48 | 1.07 | 0.104 |
| Gag vs Non-gag | 892 | -0.272 | 0.762 | 0.076 | 7.627 | 0.817 |
| DNA.VV vs DNA | 892 | 6.688 | 802.805 | 280.001 | 2301.758 | <0.001 |
| Protein vs DNA | 892 | 1.083 | 2.955 | 0.207 | 42.119 | 0.424 |
| VV vs DNA | 892 | 3.786 | 44.09 | 24.429 | 79.574 | <0.001 |
