# Supplementary material for: Cross-protocol assessment of induction and durability of VISP/R in HIV preventive vaccine trial participants
Source: PLOS Glob Public Health. 2023 Jun 8;3(6):e0002037. doi: 10.1371/journal.pgph.0002037 (PMC10249892; doi:10.1371/journal.pgph.0002037)
Supplement: S3 Table — (DOCX) [file pgph.0002037.s005.docx]

**S3 Table: HVTN 910 Demographics**

|  | Sub-Saharan Africa | | Americas/EuropeWP | |
| --- | --- | --- | --- | --- |
|  | N = 230 | | N = 863 | |
|  | N | % | N | % |
| Age |  |  |  |  |
| 30 or above | 39 | 17% | 336 | 39% |
| Less than 30 | 191 | 83% | 527 | 61% |
| Sex assigned at birth |  |  |  |  |
| Unknown | 2 | 1% | 3 | 0% |
| Female | 127 | 55% | 288 | 33% |
| Male | 101 | 44% | 572 | 66% |
| Race |  |  |  |  |
| Asian | 0 | 0% | 29 | 3% |
| Black or African American | 183 | 80% | 91 | 11% |
| Hispanic or Latino | 0 | 0% | 5 | 1% |
| Multiracial | 0 | 0% | 83 | 10% |
| Native Hawaiian or Other Pacific Islander | 0 | 0% | 1 | 0% |
| Unknown | 45 | 20% | 43 | 5% |
| White | 2 | 1% | 611 | 71% |
| Ethnicity |  |  |  |  |
| Hispanic | 0 | 0% | 112 | 13% |
| Non-Hispanic | 124 | 54% | 750 | 87% |
| Unknown | 106 | 46% | 1 | 0% |
| Study Phase |  |  |  |  |
| Phase 1-2a | 169 | 73% | 553 | 64% |
| Phase 2b | 61 | 27% | 310 | 36% |
