# Supplementary material for: Cross-protocol assessment of induction and durability of VISP/R in HIV preventive vaccine trial participants
Source: PLOS Glob Public Health. 2023 Jun 8;3(6):e0002037. doi: 10.1371/journal.pgph.0002037 (PMC10249892; doi:10.1371/journal.pgph.0002037)
Supplement: Attachment — Submitted filename: Response_to_Reviewers.docx [file pgph.0002037.s006.docx]

Manuscript Number: PGPH-D-22-02087

Response to Reviewers

Dear Dr. Schaefer,

We are grateful for the opportunity to submit a revised version of our manuscript (“Cross-protocol assessment of induction and durability of VISP/R in HIV preventive vaccine trial participants”) for publication in *PLOS Global Public Health*. We further appreciate the thoughtful and detailed suggestions provided by the reviewers and agree that updates have made the manuscript stronger. In making these updates we recognized additional nominal changes that we believe also improved clarity and/or helped the manuscript conform to PLOS guidelines. Per the request for resubmission letter, in addition to “clean” and “tracked-changes” versions of the manuscript, we herein present a point-by-point summary of our modifications (responses are in blue, indented at 0.5”):

Reviewer 1:

1. *Line 36-39: It’s not clear which vaccination arms/ study products are being compared.
   a) gp140+ env gene insert or protein Vs gp120 env or protein components?
   b) gp140+ env gene insert or protein Vs participants who received no env?
   c) Vs gp120 env or protein components Vs participants who received no env?*

The text has been updated to identify comparisons more clearly. Specifically, “[r]ecipients of viral vectors, protein boosts, and combinations of DNA and viral-vectored vaccines had higher odds of VISP/R compared to those who received DNA-only vaccines (odds ratio, OR = 10.7, 9.1, 6.8, respectively, p<0.001). Recipients of gp140+ env gene insert (OR = 7.079, p<0.001) or gp120 env (OR = 1.508, p<0.001) had higher odds of VISP/R compared to those participants who received no env. Recipients of gp140 protein had higher odds of VISP/R than those that did not receive protein (OR = 25.155, p<0.001), and recipients of gp120 protein, had lower odds of VISP/R than those that did not receive protein (OR = 0.192, p<0.001).”

2. *Lines 46-47: The sentence would read better if it started with “Nearly 40 years after the discovery of HIV, the virus continues to disproportionately burden vulnerable countries and groups ….…*

The text has been updated to include this suggestion.

3. *Line 48…add two words;
   a) that before 1.5 million
   b) occurred before in 202*

The text has been updated to include this suggestion.

4. *Line 113: define CDC since it is the first time it appears in the manuscript.*

This abbreviation is now properly introduced.

5. *To improve clarity, I would suggest moving the text between lines 118-127 to appear under the description of the cohort. Merge the information in that paragraph with the cohort description*

The text has been reworked slightly to include this suggestion.

6. *Lines 133-137: Since a total of 8,155 participants were tested for VISP/R out of 21,578 participants initially included in the analysis, it would be good to know how many of the 21,578 were placebo recipients, how many acquired HIV infection during follow up and how many did not complete the studies. This would help to understand why only about a third of the participants were tested for VISP/R at the end of their parent protocols.*

The text has been updated to include the number of participants excluded for the listed reasons.

7. *The text in lines 140-145 does not match with what is presented in Fig 2 (the flow diagram). Fig 2 shows that 4290 participants had evidence of VISR but of these only 1146 were enrolled into HVTN910. What happened to the 3144 participants (4290-1146)? Why weren’t they enrolled into HVTN910? Moreover, the 4290 are not mentioned anywhere in the cohort description, they just pop up in Fig 2.*

The text has been modified to clarify that the referenced 3,144 participants did not consent to enroll into HVTN 910 or were not eligible for enrollment based on prespecified enrollment criteria described in the manuscript. These participants were given HIV and VISP/R testing if requested, but their data was not included in further analysis.

8. *Lines 148-152: The fig 2 legend does not match the information in fig 2. The AVEG, HIVNET and HVTN 032, 034, 050, 057, 077, 502, 503 participants are not shown in Fig 2.*

This figure and figure legend have been modified to include the AVEG and HVTN participants enrolled in HVTN 910 that did not have a previous VISP/R assessment. The HIVNET protocol was removed from the manuscript as it is co-listed as an HVTN protocol.

Also, please note that with the removal of original Figure 1, the above referenced figure is now Figure 1 (not Figure 2).

9. *Line 156: DAIDS and GCLP appear for the first time in the manuscript. They need to be defined.*

These abbreviations are now properly introduced.

10. *Line 160: CTU appears for the first time in the manuscript. They need to be defined.*

This abbreviation is now properly introduced.

11. *Line 168; FDA and CE appear for the first time in the manuscript. They need to be defined.*

These abbreviations are now properly introduced.

12. *Table 3: The probability of developing VISP/R is not related to participant characteristics. It’s highly influenced by the vaccine characteristics and the immunization schedule. I wonder why participants’ results have been split into two groups; sub-Saharan Africa vs the rest of the World. It would be good if the authors had provided justification for splitting the analysis of the data into those two groups. Additionally, I would suggest the authors add a column for overall (combined) results in the table, so the reader sees an overall picture first before going into the subgroups.*

A new first column has been included to show the overall (combined) results. Regarding the rationale for the groups, HIV diagnostic testing was performed regionally, and specimens from participants enrolled in Europe, the Americas, and WP were tested on comparable diagnostic platforms (this is noted in the Statistical Analysis section). We therefore grouped these participants into one region to adjust for effects of regional differences in diagnostic tests in use.

13. *Table 4: add “s” to the word odd in the column containing multivariate odd ratio*

This has been corrected in the table.

14. *Line 262: Add “the” before risk of VISP/R by binding antibody titers to Env*

The text has been updated to include this suggestion.

15. *Line 287: EOS appears for the first time. It should be defined.*

We ended up opting for different language to prevent confusion, EOS was meant to be evaluation of seroreactivity, but has consistently been replaced with “VISP/R assessment”.

16. *Table 5:
    a) Provide overall (combined) results first*

This suggested column has been added.

*b) Provide justification for splitting the results into two groups*

See response to comment #12.

*c) There should be an explanation below the table explaining why for some parameters the total number of volunteers is less than 1093 (i.e 1085 and 1084)*

This has been clarified in an added footnote of the table.

*d) HVTN910 should NOT be written as 910*

This has been corrected.

*e) Median years since 910 enrollment [IQR].. what does it stand for? follow-up time since enrollment in HVTN 910? It’s not clear*

This has been clarified in the table and made consistent with other similar rows. Specifically, IQR is replaced with [25%, 75%].

*f) Median years since 910 enrollment (%)… same comment as above*

This has been clarified in the table and made consistent with other similar rows.

*g) What is the difference between 0 and 0-1? Why shouldn’t the authors present the data as <1 year?*

We understand the confusion in presentation here; 0 represents only one VISP visit with no follow-up, whereas 0-1 indicates some level of follow-up, but less than one year. We have included a footnote with this explanation.

*h) The content would be clearer if both actual number and % were used in the table 5 i.e 4 (X%).*

Table 5 and Table 3 now both now give percentages and actual numbers.

*i) Years since EOS [IQR]… Same comments as in e above*

This has been clarified in the table and made consistent with other similar rows.

*j) Years since last vaccination [IQR]… Same comments as in e above*

This has been clarified in the table and made consistent with other similar rows.

*k) Median N of VISP assessment. what does N mean? Also add (IQR)*

“N” has been replaced with “number”, and “[25%, 75%]” was added to indicate the presence of the IQR consistent with other rows.

*l) Three consecutive Ns over 1 yr… What does Ns and Yr mean?*

“N” has been replaced with “negative” and “Yr” has been replaced with year.

*m) Ensure uniformity in presentation of data. The numbers and percentages in the table should either be centred, aligned to the right or to the left.*

Formatting has been made consistent across tables.

17. *Lines 308-334: Maintain consistency. Indicate 95% CI inside the parenthesis of all VISP/R results. i.e 63% (95% CI: 46-86%), 23% (18-29%), 69% (64-76%), 97% (90-100%).*

The additional “95% CI:”s have been inserted for consistency.

18. *Lines 353-355: it’s written that the study “found that some HIV vaccine candidates have induced VISP/R in study participants lasting between 10-30 years”. But the participants were only followed up for a maximum of 10 years only. The authors should rephrase the statement to reflect the results in their manuscript.*

We appreciate that the 30-year figure has caused some confusion among both reviewers. We have attempted to clarify that for the formal analysis, participants were censored at 10 years, but our study population did include AVEG participants from studies as early as 1992 that were still seroreactive. Thus, while more of an “observation” than a “finding” we reference the 30-year figure to emphasize the potential for persistence of VISP/R. We have tried to clarify this in the text.

19. *Line 407: an ‘a’ is missing in the word metanalysis.*

The text has been updated to “[m]eta-analysis”

Reviewer 2:

1. *Figure 1: Sorry to say that it is not very informative. The right area of the graph with the rapid test image does not describe the VISP algorithm and is quite confusing.*

Upon review, we too recognize that Figure 1 is lacking in contribution and clarity to the manuscript. It has been removed and other figure numbers have been updated accordingly.

2. *Line 106: Do the authors mean that gp120 and gp41 were grouped together? Please specify as the groupings are not apparent in Table 1.*

The text in the Methods has been updated to clarify these groupings.

3. *Line 119: Unscheduled testing, but when were they recommended ?*

The text has been updated to indicate that testing was recommended approximately every six months.

4. *Line 179: Antibody titer data appear at this point in the manuscript. What are the objectives in this study? Please discuss in the Introduction and briefly justify the choice of specific IgG antibodies instead of just giving references.*

We clarified that available total IgG BAMA data was reanalyzed in this study for the purpose of characterizing the relationship between vaccine induced antibodies and antibody reactivity to HIV diagnostic tests. Text was removed from the Laboratory Testing section, as no new laboratory assays were performed.

5. *Line 244-245: A single p-value does not allow to conclude that the 3 platforms are different from DNA platform. My suggestion is to add the confidence intervals of the multivariate odds ratios in Table 4.*

The suggested confidence intervals have been added to the table.

6. *Line 256: OR=1.634 in the text but OR=0.752 in SI Table 1. Please clarify.*

We thank the reviewer for catching this mistake, 1.634 was correct. We also note that upon reviewing this mistake it was noted that the OR value for Region was also incorrect (it has been corrected from 2.559 to 4.478).

7. *Figure 3: Gray circles are outside 1.5 x interquartile range. Please check the legend.*

We thank the reviewer for noting this error in the legend and we have corrected it.

8. *Table 5: For times since vaccination and since EOS, the 4+ year class is not very informative. We would have seen more extreme classes and the range in addition to the IQR.*

We appreciate there were general issues in the interpretation of Table 5 and have made various adjustments. We added the min and max values in addition to the IQR, as suggested, as well as changed the bins on the year range to include “≥4, <10” and “≥10, <30”. We struggled to implement any other specific adjustments based on this comment. We are hopeful these and other modifications in Table 5, as well as updates elsewhere in the manuscript have helped clarify.

9. *Line 354: ‘10-30 years of VISP/R’, this is not shown in the results (see Table 5 comment)*

As noted in response #18 to Reviewer #1, we appreciate that the 30-year figure has caused some confusion. We have attempted to clarify that for the formal analysis, participants were censored at 10 years, but our study population did include AVEG participants from studies as early as 1992 that were still seroreactive. Thus, while more of an “observation” than a “finding” we reference the 30-year figure to emphasize the potential for persistence of VISP/R. We have tried to clarify this in the text.

10. *Line 369: ‘98% no longer had VISP/R 10 years after” , not in the Results , except in Figure S1 but only graphically*

This statistic does appear in the results, however, it was referenced as 2% persistence. We appreciate that this may have caused confusion and have updated the text in the results and the discussion to better maintain consistency.

11. *Figure S2: Individual points are beyond the “outliers” in Figure 3. Please check or justify.*

Figure S2 was meant to show a lack of data in some groups, however, we recognize in its current state it does not serve this purpose and does not add to the clarity of the manuscript. The perceived discrepancy in outliers noted was a result of points being distributed in areas that were not above the corresponding boxplots. Therefore, the outliers presented in the similar Figure 3 are correct, and since it still presents the desired message, we believe it best to remove Figure S2 in it’s current state. Figure S3 is now labeled Figure S2 and the text has been updated to reflect this. If it is specifically desired, we would be willing to try to generate a new and improved version of the old Figure S2.
